# SIRT1 suppresses adipogenesis by activating Wnt/β-catenin signaling in vivo and in vitro

**DOI:** 10.18632/oncotarget.12774

**Published:** 2016-10-20

**Authors:** Yuanfei Zhou, Tongxing Song, Jie Peng, Zheng Zhou, Hongkui Wei, Rui Zhou, Siwen Jiang, Jian Peng

**Affiliations:** ^1^ State Key Laboratory of Agricultural Microbiology, Division of Animal Infectious Disease, College of Veterinary Medicine, Huazhong Agricultural University, Wuhan, P. R. China; ^2^ Department of Animal Nutrition and Feed Science, College of Animal Science and Technology, Huazhong Agricultural University, Wuhan, P. R. China; ^3^ Key Laboratory of Swine Genetics and Breeding of Agricultural Ministry, and Key Laboratory of Agricultural Animal Genetics, Breeding and Reproduction of Ministry of Education, College of Animal Science and Technology, Huazhong Agricultural University, Wuhan, P. R. China; ^4^ The Cooperative Innovation Center for Sustainable Pig Production, Wuhan, P. R. China

**Keywords:** mesenchymal stem cells, adipogenesis, SIRT1, Wnt signaling antagonists, β-catenin

## Abstract

Sirtuin 1 (SIRT1) regulates adipocyte and osteoblast differentiation. However, the underlying mechanism should be investigated. This study revealed that SIRT1 acts as a crucial repressor of adipogenesis. RNA-interference-mediated SIRT1 knockdown or genetic ablation enhances adipogenic potential, whereas SIRT1 overexpression inhibits adipogenesis in mesenchymal stem cells (MSCs). SIRT1 also deacetylates the histones of *sFRP1*, *sFRP2*, and *Dact1* promoters; inhibits the mRNA expression of *sFRP1*, *sFRP2*, and *Dact1*; activates Wnt signaling pathways; and suppresses adipogenesis. SIRT1 deacetylates β-catenin to promote its accumulation in the nucleus and thus induces the transcription of genes that block MSC adipogenesis. In mice, the partial absence of *SIRT1* promotes the formation of white adipose tissues without affecting the development of the body of mice. Our study described the regulatory role of SIRT1 in Wnt signaling and proposed a regulatory mechanism of adipogenesis.

## INTRODUCTION

Adipocytes originate from mesenchymal stem cells (MSCs) that also function as precursors of muscle, cartilage, and bone cells [1]. Adipogenesis can be divided into two stages, namely, commitment and terminal differentiation [2, 3]. Cell differentiation from pre-adipocytes to adipocytes is regulated by transcription factors. In terminal differentiation, CCAAT/enhancer-binding protein α (C/EBPα) and nuclear receptor peroxisome proliferator-activated receptor γ (PPARγ) are considered two key transcription factors because they enhance gene promoter activities essential for adipocyte phenotype [4]. However, these aspects have yet to be fully elucidated. Transcriptional regulatory networks in commitment differentiation have also yet to be fully established although some transcription factors [5, 6] and signaling pathways have been confirmed [3]. As such, specific factors and molecular mechanisms involved in determination should be further investigated.

The activation of the Wnt/β-catenin signaling pathway follows an inverse pattern between the induction of MSC osteogenic differentiation and the induction of MSC adipogenic differentiation. Wnt/β-catenin signaling elicits a switching effect on osteogenic stimulation during adipogenic inhibition [7, 8]. This signaling pathway is also inhibited by three factors: extracellular Dickkopf (Dkk), which sequesters LRP5/6 co-receptors; extracellularly secreted frizzled related proteins (sFRPs), which separate Wnt ligands; and intracellular Dapper (Dact), which binds to disheveled (Dvl) proteins [9]. Although research has demonstrated that Wnt antagonists play a crucial role in the differentiation of pre-adipocytes to mature adipocytes [10], studies have been rarely performed to describe the commitment of Wnt antagonists. The constitutive expression of *sFRP1 in 3T3-L1 pre-adipocytes promotes adipogenesis and lipid accumulation* [10], but Dact1 knockdown impairs adipogenesis by activating the Wnt/β-catenin signaling pathway in 3T3–L1 pre-adipocytes [11]. Nevertheless, the mechanism by which sFRPs and Dacts are regulated during adipogenesis remains unknown.

Sirtuin 1 (SIRT1) is a NAD^+^-dependent lysine deacetylase involved in multiple cellular events, including transcriptional silencing, cell proliferation, differentiation, senescence, apoptosis and metabolism, stress responses, lifespan control, glucose homeostasis, insulin secretion, adipocyte differentiation, and metabolism [12, 13]. SIRT1 is also involved in adipogenesis [12]. For instance, SIRT1 activation inhibits adipocyte development and enhances the expression of osteoblast markers; by contrast, SIRT1 inhibition increases the number of adipocytes and promotes the expression of adipocyte markers in C3H10T1/2 [14]. SIRT1 is activated by resveratrol to inhibit adipogenic differentiation and improve myogenic differentiation; conversely, SIRT1 is inhibited by nicotinamide-promoted adipogenic differentiation [15]. In MSC-specific SIRT1-knocked out mice, the weight of subcutaneous fat decrease with small adipocytes and the concentration of blood triglycerides increase [16]. In SIRT1 null (*SIRT1*^−/−^) mice, the average adipocyte size is small, the extracellular matrix content is reduced, the expression levels of adiponectin and leptin are 60% of the normal level, and the differentiation of adipocytes in adipose tissues is reduced [17]. SIRT1 may also interact with Wnt signaling in some biological events. Holloway et al. (2010) found that SIRT1 promotes constitutive Wnt signaling and Wnt-induced cell migration in several cancer cells [18]. SIRT1 activation by resveratrol promotes osteoblastic differentiation by stabilizing and stimulating the nuclear accumulation of β-catenin in ST2 cells [19]. SIRT1 deacetylates β-catenin and promotes its nuclear localization and activity; consequently, genes involved in MSC differentiation become transcribed [16]. SIRT1 also promotes myoblast cell proliferation by partly stimulating the Wnt signaling pathway in C2C12 cells [20]. However, the interaction between SIRT1 and Wnt signaling in the regulation of the commitment of MSCs to the adipose lineage remains largely unknown.

Our study aimed to determine whether SIRT1 inhibits the commitment of MSCs to the adipocyte lineage by activating Wnt signaling and to elucidate the underlying molecular mechanisms. Our findings may provide insights into fat formation and adipogenesis regulatory network.

## RESULTS

### Reducing SIRT1 enhances adipogenesis *in vitro*

**Figure 1:**
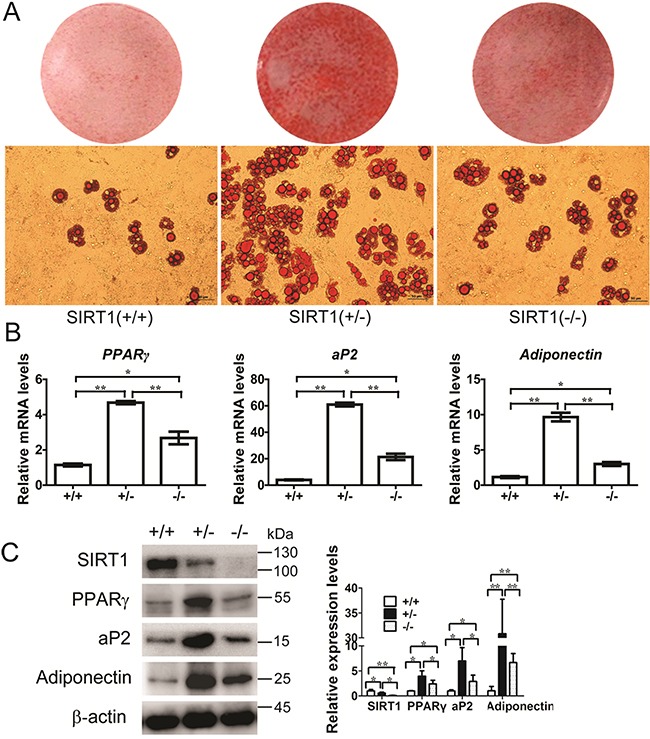
SIRT1 inhibits adipogenesis in MEFs **A.** MEFs were prepared from a 13.5-d embryo and induced for adipogenesis in the culture medium. Oil red O staining for triglyceride in differentiated MEFs for 8 days with the standard differentiation protocol. n = 6 wells per group. **B.** The mRNA expression of adipocyte markers (*PPARγ*, *aP2* and *adiponectin*) were detected by Real-time PCR at day 8. Bars indicate SD. *P < 0.05; **P < 0.01. n = 3 per group. **C.** The proteins expression of adipocyte markers (PPARγ, aP2 and adiponectin) were detected by immunoblotting at day 8. Bars indicate SD. *P < 0.05; **P < 0.01. n = 3 per group.

Wild-type (WT) *SIRT1*^+/+^, *SIRT1*^+/−^, and *SIRT1*^−/−^ mouse embryonic fibroblasts (MEFs) were isolated and induced with an adipogenic medium to identify whether SIRT1 inhibits the commitment of MSCs to the adipocyte lineage. Compared with those of the WT cells, lipid accumulation in the cells differentiated from *SIRT1*^+/−^ and *SIRT1*^−/−^ MEFs were enhanced, as observed by oil red O staining. The lipid accumulation in *SIRT1*^+/−^ MEFs (Figure 1A) was greater than that in the other cells. This finding suggested that adipogenic potential increased. After completing differentiation, the *SIRT1*-deficient cells showed a significant increase in the mRNA and protein levels of PPARγ, ap2, and adiponectin (Figures 1B and 1C). This result was more evident in the *SIRT1*^+/−^ MEFs than in the other cell types (Figures 1B and 1C). These observations indicated that SIRT1 acts as a repressor of adipogenesis in MEFs.

### SIRT1 activates Wnt/β-catenin signaling pathway activity

**Figure 2:**
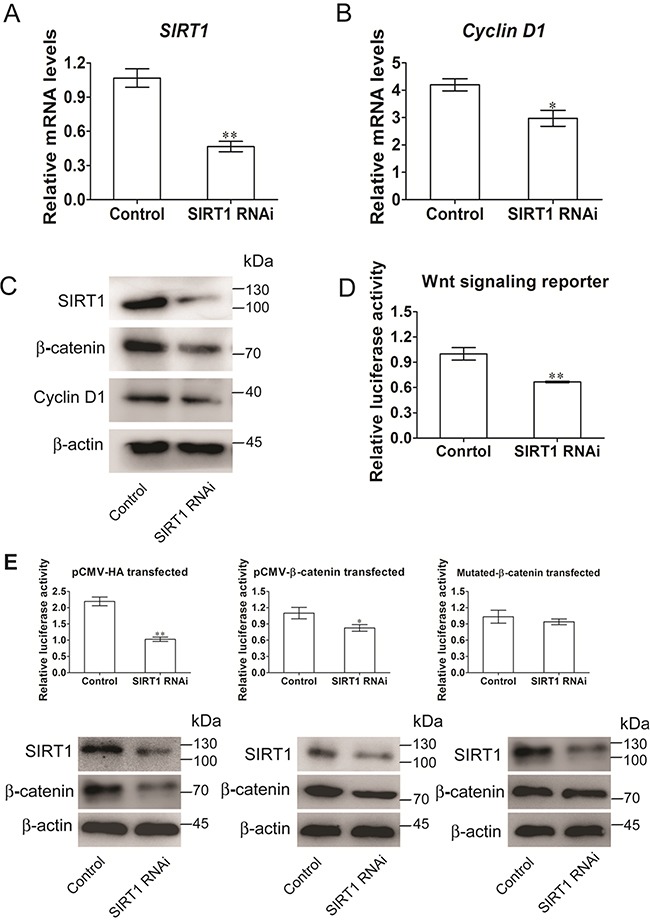
SIRT1 stimulates Wnt/β-catenin signaling in C3H10T1/2 cells **A.** The mRNA levels of *SIRT1* were measured 48h after transfection. Bars indicate SD. **P < 0.01. n = 3 per group. **B.** The mRNA levels of Wnt signalling pathway of target gene *Cyclin D1* was measured by Real-time PCR at 48h after transfection. Bars indicate SD. *P < 0.05. n = 3 per group. **C.** The protein levels of SIRT1, Cyclin D1 and β-catenin were measured by immunoblotting at 48h after transfection. **D.** TOPflash/FOPflash was co-transfecred with siRNA of *SIRT1 in C3H10T1/2 cells. The luciferase reporter activity was measured at 24h after transfection. Bars indicate SD.* **P < 0.01. n = 3 per group. **E.** C3H10T1/2 cells were co-transfecred with TOPflash/FOPflash, control or siRNA of *SIRT1* and with pCMV-HA (control), or pCMV-catenin or pCMV-catenin mutation vector. The luciferase reporter activity was measured at 24h after transfection, and β-catenin were measured by immunoblotting. Bars indicate SD. *P < 0.05; **P < 0.01. n = 3 per group.

RNA interference (RNAi) was initially applied to disrupt the SIRT1 function and to determine whether SIRT1 activates the Wnt/β-catenin signaling pathway during the commitment of MSCs to the adipocyte lineage. The *SIRT1* RNAi plasmid was used to infect C3H10T/2 cells, and the interference efficiency was examined (Figure 2A). The mRNA expression of *Cyclin D1*, a target gene of the canonical Wnt pathway, was significantly reduced when SIRT1 was knocked down by RNAi (Figure 2B). The protein levels of SIRT1, cyclin D1, and β-catenin, which are key mediators of Wnt signaling, were also reduced significantly (Figure 2C). We also examined whether SIRT1 activates Wnt/β-catenin signaling by subjecting C3H10T1/2 cells to a TOP/FOP reporter assay. The RNAi expression of *SIRT1* inhibited luciferase activation by 40% (Figure 2D). To determine whether SIRT1 activates the Wnt signaling pathway via the β-catenin-dependent canonical pathway, we co-transfected the WT or mutant β-catenin with pSIREN-*SIRT1* and TCF-reporter. In mutant β-catenin, the GSK3β phosphorylation sites S33, 37, and 45A T41A were mutated. Luciferase assay revealed that the SIRT1 knockdown blocked Wnt/β-catenin signaling in early stages 24 h after transfection. By contrast, the inhibitory activity was disrupted by the mutant β-catenin, which resisted proteasomal degradation (Figure 2E). The protein expression of β-catenin exhibited a similar pattern (Figure 2E). These results implied that SIRT1 may promote Wnt/β-catenin signaling activity.

### SIRT1 inhibits the expression of Wnt signaling antagonists

**Figure 3:**
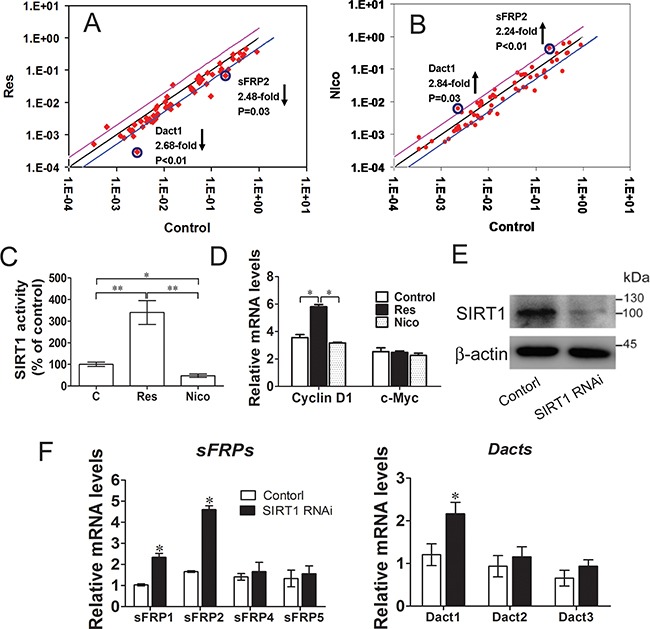
SIRT1 suppresses Wnt signaling antagonists **A.** C3H10T1/2 Cells were treated with 25 mmol/L of resveratrol for 12h, the relatively gene of Wnt signalling were meansured by Real-time PCR Array. Lines below of the center regression lines indicate 2-fold changes in gene expression. n = 3 per group. **B.** C3H10T1/2 Cells were treated with 10mmol/L nicotinamide for 12h, the relatively gene of wnt signaling were meansured by Real-time PCR Array. Lines above of the center regression lines indicate 2-fold changes in gene expression. n = 3 per group. **C.** The SIRT1 activity was determined using a SIRT1 fluorometric assay kit at 12h after treatment with resveratrol or nicotinamide. *P < 0.05; **P < 0.01. n = 3 per group. **D.** Wnt signalling target genes (*Cyclin D1* and *c-Myc*) mRNA levels were meansured by Real-time PCR at 12h after treatment with resveratrol or nicotinamide. *P < 0.05. n = 3 per group. (E-F) C3H10T1/2 cells were infected with shRNA specific for SIRT1 (SIRT1 RNAi), or a scrambled hairpin (Control). **E.** The protein levels of SIRT1 were measured by immunoblotting. **F.** Wnt signalling antagonists (*sFRPs* and *Dacts*) mRNA levels were meansured by Real-time PCR. n = 3 per group.

We used real-time quantitative PCR arrays to screen the SIRT1-regulated Wnt pathway signaling components and to obtain additional evidence showing that SIRT1 activates the Wnt signaling pathway. We performed these experiments in both gain-of-function and loss-of-function models to identify the actual targets of SIRT1 and focused on the genes with coordinating regulatory mechanisms between SIRT1 activation and inactivation. These results were confirmed by the quantitative PCR profiling of Wnt target genes (Supplementary Tables S5 and S6). Interestingly, *sFRP2* and *Dact1* were inhibited in resveratrol-treated C3H10T1/2 cells (Figure 3A), whereas *sFRP2* and *Dact1* were promoted in nicotinamide-treated C3H10T1/2 cells (Figure 3B). These results showed that *sFRP2* and *Dact1* genes were coordinately changed. Resveratrol treatment significantly increased SIRT1 activity, and nicotinamide treatment reduced SIRT1 activity, as revealed by SIRT1 deacetylase fluorometric assay (Figure 3C). The mRNA levels of *cyclin D1* were also increased in resveratrol-treated cells, whereas the mRNA levels of *c-Myc* remained unchanged in different treatments (Figure 3D). SIRT1 knockdown reduced the levels of ectopically expressed SIRT1 (Figure 3E). Conversely, the mRNA levels of *sFRP1*, *sFRP2*, and *Dact1* were enhanced (Figures 3F). These results suggested that SIRT1 promotes Wnt/β-catenin signaling by inhibiting the expression of sFRP1, sFRP2, and Dact1 of Wnt signaling antagonists.

### SIRT1 represses Wnt signaling antagonists during adipogenesis

**Figure 4:**
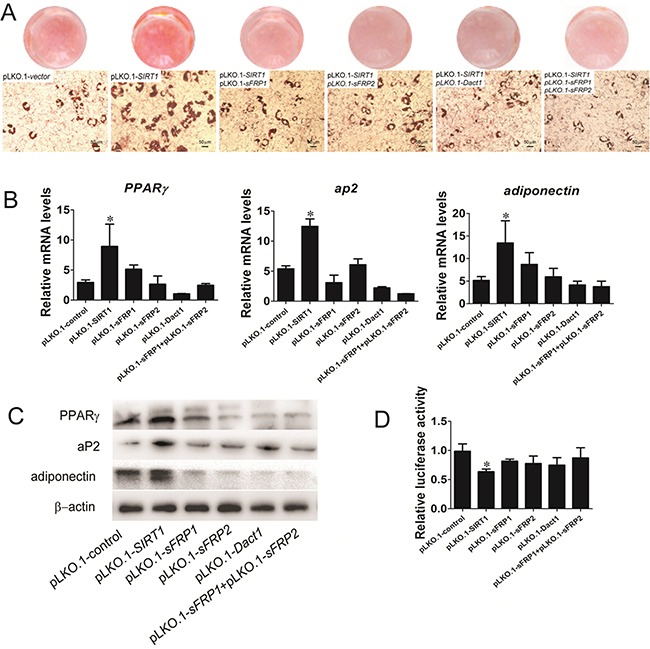
SIRT1 represses adipogenesis by blocking Wnt signaling antagonists **A.** Oil red O staining for triglycerides in infected C3H10T1/2 cells differentiated for 8d. **B.** The infected C3H10T1/2 cells were induced by using standard differentiation protocol. A: The mRNA expression of adipocyte markers (*PPARγ*, *aP2* and *adiponectin*) were detected by Real-time PCR at day 8. Bars indicate SD. *P < 0.05. n = 3 per group. **C.** The protein expression of adipocyte markers (PPARγ, aP2 and adiponectin) were detected by immunoblotting at day 8. **D.** TOPflash/FOPflash was transfecred in infected C3H10T1/2 cells. The luciferase reporter activity was measured at 24h after transfection. Bars indicate SD. *P < 0.05. n = 3 per group.

We initially used lentivirus to generate stable C3H10T1/2 cell lines and to evaluate the role of SIRT1 in pre-adipocyte commitment by regulating Wnt signaling antagonists. The stable transfected cells were subjected to adipogenic differentiation, and the samples were collected for adipogenic analysis. The fat droplet was increased in the SIRT1-knocked down cells by infecting with the pLKO.1-*SIRT1* (Figure 4A). Compared with the SIRT1-knocked down cells, the stable cells downregulated SIRT1 and Wnt signaling antagonists through RNAi vector co-infection and accumulated much less fat droplets, as determined by oil red O staining (Figure 4A). The stable cells also presented reduced mRNA expression (Figure 4B) and protein levels (Figure 4C) of the adipogenic marker gene PPARγ, ap2, and adiponectin. Wnt signaling was also analyzed through TCF luciferase reporter assay. The Wnt reporter activity was reduced in the stable cells infected with the downregulated SIRT1 and Wnt signaling antagonist RNAi vectors (Figure 4D). These data indicated that SIRT1 repressed adipogenesis by blocking Wnt signaling antagonists.

### SIRT1 regulates histone acetylation at the promoter sites of Wnt signaling antagonists

**Figure 5:**
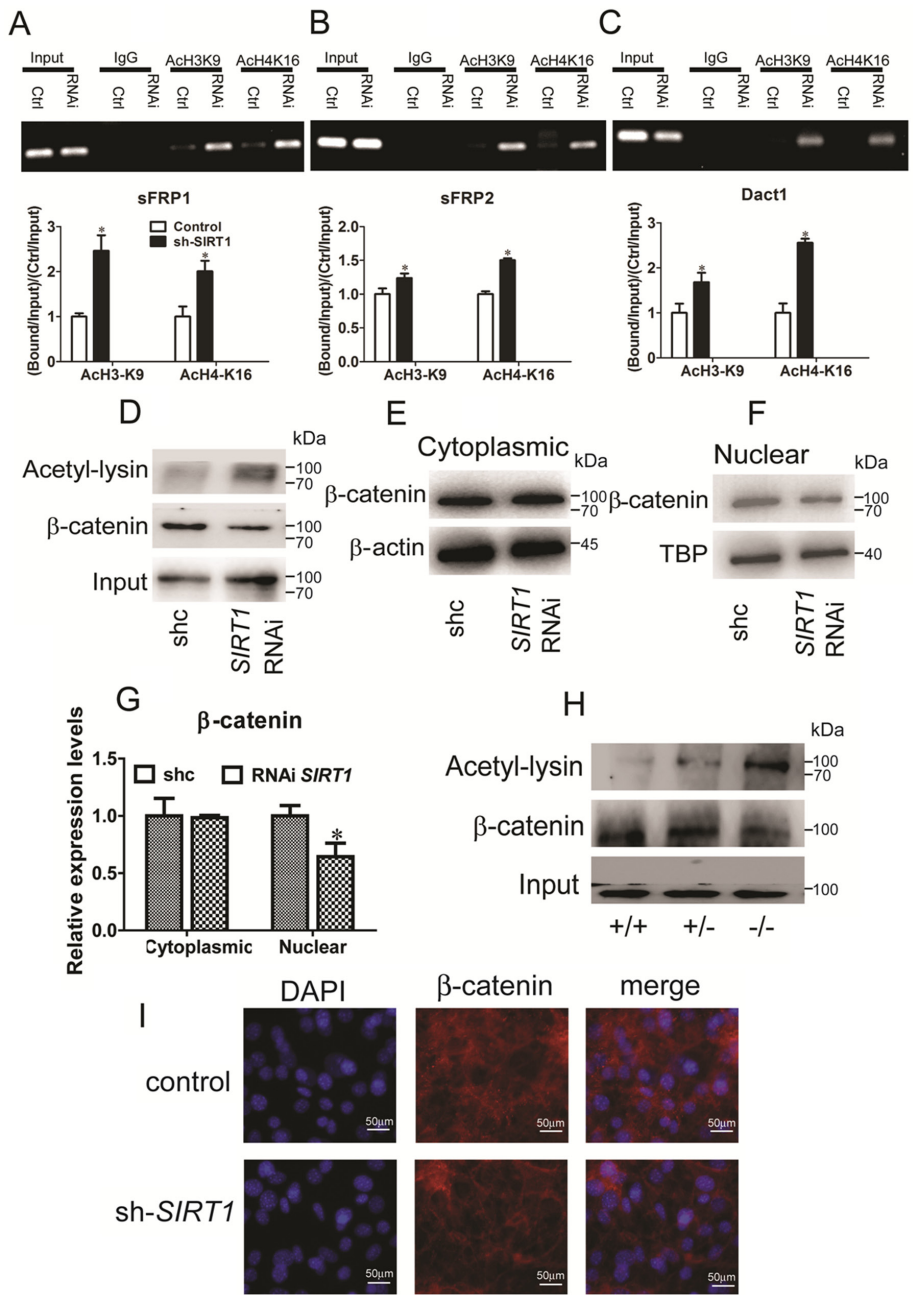
SIRT1 regulates Wnt signaling by deacetylating histones and β-catenin **A, B** and **C.** Pooled populations of C3H101/2 cells stably selected to express *SIRT1* RNAi constructs were analyzed via ChIP. ChIP was performed with antibodies acetylated histone H3, lysine 9 (H3K9) and H4, lysine 16 (H4K16), or with IgG antibody controls. Promoter sequence was amplified by RT-PCR under linear conditions for the genes *sFRP1* (A), *sFRP2* (B) and *Dact1* (C). The acetylation of H3K9 and acetylation of H4K16 at the *sFRP1* (A), *sFRP2* (B) and *Dact1* (C) promoters were measured by Real-time PCR. Bars indicate SD. *P < 0.05. n = 3 per group. **D.** After stably transfected with *SIRT1* RNAi plasmid in C3H101/2 cells. Endogenous β-catenin was immunopreciptated with anti-β-catenin, and acetylated β-catenin was identified using anti-acetyl-lysine. **E.** The protein levels of β-catenin in cytoplasms were measured by immunoblotting. **F.** The protein levels of β-catenin in nuclear were measured by immunoblotting. **G.** The relative expression protein levels of β-catenin in cytoplasmic and nuclear. Bars indicate SD. *P < 0.05. n = 3 per group. **H.** Pooled populations of of differentiated MEF cells were analyzed via IP. β-catenin was immunopreciptated with anti-β-catenin, and acetylated β-catenin was identified using anti-acetyl-lysine. **I.** Immunofluorescent staining of DAPI and β-catenin in control and *SIRT1* RNAi C3H101/2 cells.

To elucidate how SIRT1 contributes to the silencing of Wnt signaling antagonists, we examined the modification of lysine residues associated with transcriptional repression mapped with SIRT1-associated gene silencing. We subjected C3H10T1/2 cells and MEFs to chromatin immunoprecipitation (ChIP) assays. During the reactivation of *sFRP1* and concurrent shRNA knockdown of *SIRT1* in C3H10T1/2 cells, we observed that the acetylation of H3K9 and H4K16 increased (Figure 5A). The levels of H3K9 and H4K16 acetylation at *sFRP2* and *Dact1* promoters were also increased (Figures 5B and 5C). The levels of H3K9 and H4K16 acetylation at the *sFRP1*, *sFRP2*, and *Dact1* promoters in *SIRT1*-deficient MEFs were significantly increased (Supplementary Figures S1A, S1B, and S1C). These results indicated that SIRT1 inhibition increased the acetylation of H3K9 and H4K16, reduced their activities, and decreased the expression levels of *sFRP1*, *sFRP2*, and *Dact1*. As such, SIRT1 may suppress the activation of the Wnt signaling pathway.

### SIRT1 deacetylates and promotes the nuclear accumulation of β-catenin

Simic et al. (2013) reported that SIRT1 selectively binds to and deacetylates β-catenin and promotes its accumulation in the nucleus; as a result, genes involved in MSC differentiation are transcribed [16]. Therefore, we verified the identity of SIRT1-regulated β-catenin. We examined whether SIRT1 deacetylates β-catenin in C3H10T1/2 cells. Lysyl-acetylated β-catenin was detected by immunoblotting with anti-acetyl-lysine in immunoprecipitates by anti-β-catenin. We found that the acetylated β-catenin level was increased by SIRT1 silencing (Figure 5D). We then examined the cytoplasmic and nuclear β-catenin levels in response to SIRT1. In the cytoplasm, β-catenin expression remained unchanged (Figures 5E and 5G). In the nucleus, SIRT1 silencing reduced the accumulation of β-catenin (Figures 5F and 5G). Additionally, the acetylated β-catenin levels in the *SIRT1*-deficient MEFs (*SIRT1*^+/−^ and *SIRT1*^−/−^ MEFs) were increased (Figure 5H). We also observed the β-catenin localization in the *SIRT1*-knocked down C3H10T1/2 cells with immunofluorescence. The RNAi expression of *SIRT1* and the nuclear accumulation of β-catenin were lower than those in the control group (Figure 5I). Indeed, SIRT1 directly binds to and deacetylates β-catenin and increases its accumulation in the nucleus. As a consequence, related genes are transcribed through the regulation of the Wnt signaling pathway.

### SIRT1 suppresses adipogenesis *in vivo*

**Figure 6:**
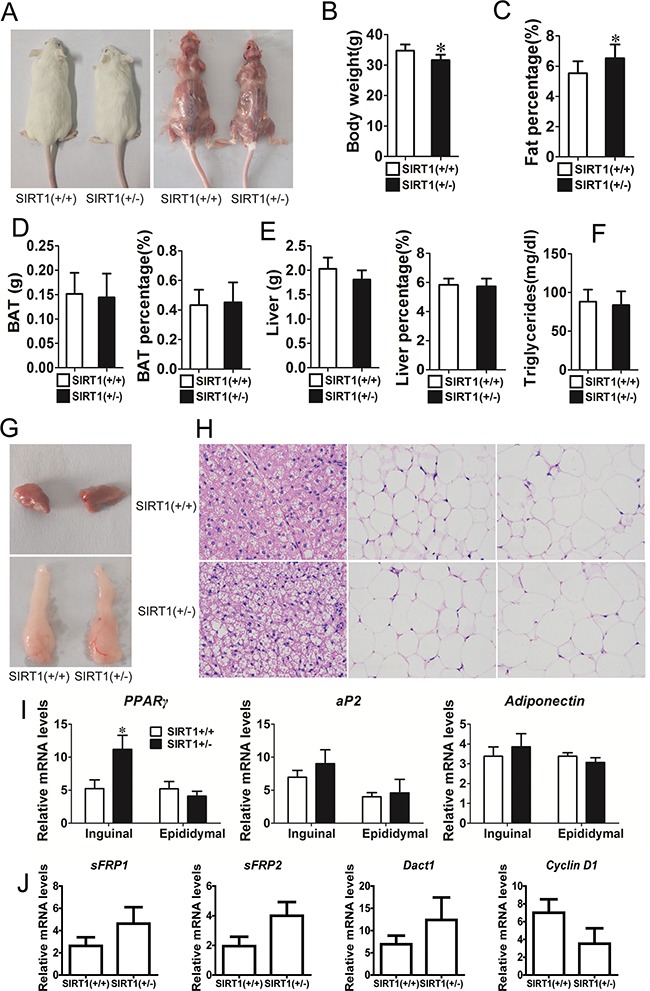
Reduction of SIRT1 enhances adipogenic potential in vivo **A.** Comparison of *SIRT1*^+/+^ and *SIRT1*^+/−^before and after killing in aged 12w mice. **B.** Body weight of *SIRT1*^+/+^ and *SIRT1*^+/−^ in aged 12w mice. Bars indicate SD, n=7. **C.** Fat mass weight relative to total body weight composition of *SIRT1*^+/+^ and *SIRT1*^+/−^ in aged 12w mice. Bars indicate SD, n=7. **D.** BAT weight, BAT weight relative to total body weight composition of *SIRT1*^+/+^ and *SIRT1*^+/−^ in aged 12w mice. Bars indicate SD, n=7. **E.** Liver weight, liver weight relative to total body weight composition of *SIRT1*^+/+^ and *SIRT1*^+/−^ in aged 12w mice. Bars indicate SD, n=7. **F.** Blood triglyceride level in *SIRT1*^+/+^and *SIRT1*^+/−^ in aged 12w mice. Bars indicate SD, n=7. **G.** BAT and WAT of epididymal fat pad. **H.** Hematoxylin and eosin staining of BAT, subcutaneous adipose tissue (inguinal) and visceral adipose tissue (epididymal) of *SIRT1*^+/+^ and *SIRT1*^+/−^ in aged 12w mice. **I**. The mRNA expression of adipocyte markers (*PPARγ*, *aP2* and *adiponectin*) were determined in inguinal and epididymal of S*IRT1*^+/+^and *SIRT1*^+/−^ mice by Real-time PCR. Bars indicate SD. *P < 0.05. n = 7. **J.** The mRNA expression of *sFRP1*, *sFRP2*, *Dact1* and *Cyclin D1* were determined in subcutaneous of S*IRT1*^+/+^ and *SIRT1*^+/−^ mice by Real-time PCR. Bars indicate SD. n = 7.

The role of SIRT1 in adipogenesis was also investigated *in vivo*. Unfortunately, the *SIRT1*^−/−^ mice succumbed to early postnatal lethality. Few mice survived but barely reached adulthood and exhibited other severe phenotypes; as such, these mice were not used in our study [21, 22]. Therefore, we focused our analysis on the development of the WT and *SIRT1*^+/−^ mice, which were phenotypically normal. The phenotype of the *SIRT1*^+/−^ mice was normal phenotype and their body size was slightly smaller than that of the *SIRT1*^+/+^ mice (Figure 6A). The body weight of the *SIRT1*^+/−^ mice was also lower than that of the *SIRT1*^+/+^ mice (Figure 6B). The fat mass of the *SIRT1*^+/−^ mice was comparable to that of the *SIRT1*^+/+^mice, but the ratio of fat mass to body weight of the *SIRT1*^+/−^ mice was markedly higher than that of the *SIRT1*^+/+^ mice (Figure 6C). This finding suggested that the fat content accumulated in the *SIRT1*^+/−^ mice. The brown adipose depots of the *SIRT1*^+/−^ mice remained unchanged in terms of tissue weight and tissue percentage (Figure 6D). Similarly, the liver weight and the ratio of liver to body weight were not altered between the two genotypes (Figure 6E). Blood triglyceride levels did not differ between the two genotypes (Figure 6F).

WAT and BAT were further investigated using inguinal fat tissue (white fat), epididymal fat tissue (white fat), and brown fat tissue in this study. Brown fat and epididymal fat were similar in the two mouse genotypes (Figure 6G). Hematoxylin and eosin staining revealed that adipocyte size and extracellular matrix did not significantly differ between *SIRT1*^+/−^ and *SIRT1*^+/+^ mice (Figure 6H). The mRNA expression levels of adipogenic marker gene *PPARγ*, *ap2*, and *adiponectin* were examined to characterize the changes in adipose tissues of the *SIRT1*^+/−^ mice. The mRNA expression of *PPARγ* was higher in the inguinal fat tissue of *SIRT1*^+/−^ mice. By contrast, the mRNA expression of *PPARγ* in the epididymal fat tissue remained unchanged (Figure 6I). The mRNA expression of *ap2* and *adiponectin* did not differ between the two genotypes (Figure 6I). We also detected the mRNA expression of *sFRP1*, *sFRP2*, *Dact1*, and *Cyclin D1* through real-time PCR. The mRNA expression levels of *sFRP1*, *sFRP2*, and *Dact1* had an increased trend, whereas the mRNA expression level of *cyclin D1* had a downward trend (Figure 6J). These observations implied that the partial absence of *SIRT1 in mice unlikely affected the physical development of mice and improved the deposition of adipose tissues.*

## DISCUSSION

Guarente et al. [23] provided the first evidence of SIRT1 as a repressor in the differentiation of pre-adipocytes and promotion of fat metabolism. They found that SIRT1 inhibits adipogenesis in 3T3-L1 cells by repressing the activity of the pro-adipogenic nuclear receptor PPARγ; in differentiated 3T3-L1 adipocytes, SIRT1 upregulation triggers lipolysis and fat loss [23]. In the early stages of lineage commitment and cell fate determination, resveratrol and isonicotinamide markedly inhibit adipocyte differentiation and promote osteoblast differentiation, whereas nicotinamide increases the number of adipocytes and the expression level of adipocyte markers [14]. Furthermore, resveratrol-activated SIRT1 in MSCs facilitates SIRT1 binding to PPARγ and consequently represses PPARγ activity [24]. SIRT1 is knocked down by antisense oligonucleotides; consequently, the SIRT1 expression in MSCs is downregulated, and this finding demonstrates that SIRT1 can functionally inhibit osteogenesis and induce adipogenesis [24]. Our previous study found that resveratrol blocks adipocyte formation and promotes myotube differentiation in the established MSC commitment and differentiation model based on 5-azacytidine, which is a DNA methylation inhibitor [15]. By contrast, nicotinamide enhances the adipogenic potential of C3H10T1/2 cells [15]. These studies implied that SIRT1-induced adipogenic suppression occurs throughout the whole process, particularly in the terminal differentiation of adipocyte and commitment differentiation of MSCs to a pre-adipocyte fate.

Lipid accumulation was significantly increased and the mRNA and protein expression levels of PPARγ, ap2, and adiponectin were promoted in *SIRT1*-deficient cells. By contrast, the adipogenic potential of *SIRT1*^+/−^ MEFs was greater than that of the other cells (Figure 1). Xu et al. (2012) reported that the mRNA and protein expression levels of PPARγ moderately increase in *SIRT1*-deficient cells, but adiponectin expression in different genotype cells remains unchanged [17]. However, these results are inconsistent with our findings. We observed that proliferation and differentiation did not occur because of the total absence of SIRT1 MEFs. SIRT1 functions as a signal in stem cell differentiation and is necessary to correctly establish specific differentiation programs during stem cell differentiation [25]. SIRT1, as a broad-spectrum deacetylation enzyme, may participate in regulating the expression of some genes related to cell growth and development.

RNAi *SIRT1* inhibited the mRNA and protein expression levels of cyclin D1 and reduced the activity of the luciferase reporter system of Wnt signaling (Figure 2). This finding indicated that SIRT1 likely activates the Wnt signaling pathway in stem cells. In a previous study, the mRNA levels of three Wnt/Dvl target genes, namely, *BMP4*, *c-Myc*, and *cyclin D1*, are significantly reduced by inhibiting SIRT1 activity in T-47D, HCT116, and MDA-MB-231 cells [18]. In our study, the mRNA expression of *c-Myc* remained unchanged. Similarly, SIRT1 overexpression or downregulation unlikely affects the mRNA level of *c-Myc* in 293T cells and HeLa cells [26]. Menssen et al. (2011) also reported that the mRNA expression of *c-Myc* remains unchanged in *SIRT1*^+/+^ MEFs and SIRT1-deficient MEFs, but the protein levels of c-Myc are lower in SIRT1-deficient MEFs than in *SIRT1*^+/+^ MEFs [27]. This phenomenon possibly occurs because SIRT1 mediates the deacetylation during the regulation of c-Myc degradation, affects the stability of c-Myc, and reduces the protein levels of c-Myc. SIRT1 activates Wnt signaling through β-catenin (Figure 2), and this finding is consistent with previous studies. The total β-catenin levels of both HCT116 and T-47D cells are also decreased by SIRT1 inhibition [18]. The transcriptional activity of β-catenin is increased by the activation of SIRT1 [19]. These data demonstrated that SIRT1 functions as an important activator of Wnt signaling.

SIRT1 repressed adipogenesis by inhibiting the mRNA expression of *sFRP1*, *sFRP2*, and *Dact1* (Figure 3). 3T3-L1 pre-adipocytes treated with sFRP1 and sFRP2 recombinant proteins exhibit considerable spontaneous adipogenesis [28]. However, the role of sFRPs in adipogenic commitment has yet to be elucidated. Dact1 promotes adipogenesis through coordinated effects on gene expression that selectively alters the intracellular and paracrine/autocrine components of the Wnt/β-catenin signaling pathway [11]. Compared with cells infected with the pLKO.1-*SIRT1* vector, the stable cells downregulated the levels of SIRT1 and Wnt signaling antagonists that blocked adipogenesis (Figure 4) and thus improved the activity Wnt signaling to some degree. SIRT1 inhibits MSC commitment and differentiation to adipocytes by increasing Wnt signaling antagonist expression and activating the Wnt signaling pathway [15]. Nevertheless, the molecular mechanisms underlying SIRT1-inhibited adipogenesis should be further investigated. Our data found that SIRT1 regulates Wnt signaling antagonists via a mechanism involving the transcriptional repression of Wnt signaling antagonists. ChIP results indicated that SIRT1 inhibition increases the expression of *sFRP1*, *sFRP2*, and *Dact1*. The reactivation of these genes may suppress the constitutive activation of the Wnt signaling pathway (Figures 5A–5C and Supplementary Figure S1). Likewise, SIRT1 localizes to the promoter of *sFRP1* and directly contributes to the aberrant epigenetic silencing of breast cancer cells [29]. Our findings revealed that SIRT1 deacetylates the histones of *sFRP1*, *sFRP2*, and *Dact1* promoters, inhibit the mRNA expression of these genes, and prevent the inhibition of Wnt signaling. Consequently, Wnt signaling pathways are activated. These occurrences thus inhibit the commitment of MSCs to the adipocyte lineage.

Various proteins including histone and non-histone [12] proteins, such as p53 [30], NF-κB [31], PPARγ [32], and members of the FOXO family [33, 34], function as SIRT1 substrates. SIRT1 downregulation increases the level of β-catenin acetylation and promotes its accumulation in the nucleus (Figures 5D–5I). SIRT1 affects MSCs by deacetylating β-catenin, which increases the potential of MSCs to differentiate to bones and fat to a less extent [16]. These findings are consistent with our results. In epithelial cells, the SIRT1 deacetylation of β-catenin causes the release of β-catenin from the nucleus [35]. This phenomenon occurs probably because epithelial and mesenchymal cells show different β-catenin localizations [36], and nuclear import and export machineries may differ in MSCs and epithelial cells [16]. β-catenin signaling is essential for cell fate determination and differentiation. Therefore, these findings suggested that SIRT1 is associated with β-catenin in MSCs.

The partial absence of *SIRT1 in adult mice unlikely affects physical development but improves adipose tissue deposition. Similarly, the body fat content of SIRT1*^+/−^ mice is increased consistently and is 30% greater than that of the WT at 12 weeks of age [37]. Picard et al. (2004) found that the epididymal WAT mass of WT mice does not significantly differ from that of *SIRT1*^+/−^ mice [23]. We examined some adipose tissues, including interscapular fat, inguinal fat, perirenal fat, and epididymal fat. Our results suggested that each mass was not different between WT and *SIRT1*^+/−^ mice, but the ratio of total fat mass to body weight was increased in the *SIRT1*^+/−^ mice.

In conclusion, our study provided evidence describing a previously unreported regulatory pathway of SIRT1 in MSC adipogenic commitment. In this mechanism, SIRT1 inhibits MSC adipogenesis via the Wnt/β-catenin pathway. This pathway involves the epigenetic regulation of key developmental genes, such as sFRP1, sFRP2, and Dact1, which are Wnt signaling antagonists, and β-catenin, which is a key component of the Wnt signaling pathway. Consistent with *in vivo* and *in vitro* experimental findings, our results indicated that SIRT1 plays a crucial role in the inhibition of adipogenesis and contributes to the establishment of Wnt/β-catenin signaling for MSC fate determination.

## MATERIALS AND METHODS

### Animal experiments

For mouse experiments, Male heterozygous knockout (*SIRT1*^+/−^) mice, and littermate wild-type (WT) mice were obtained from the Institute for Nutritional Sciences, Shanghai Institutes for Biological Sciences, the Chinese Academy of Sciences. *SIRT1*^+/−^ mice (129 /ICR background) were described previously [21]. 12-week-old adult male *SIRT1*^+/+^ and *SIRT1*^+/−^ mice were used for the present studies. The genotyping for SIRT1 was carried out as described previously. All mice were housed under 25±1°C with a 12-h light, 12-h dark cycle. Food provided was normal chow. All mice were maintained and used in accordance with the guidelines of the Institutional Animal Care and Use Committees at the Huazhong Agricultural University. 12-week-old adult male mice, weighed again, then take blood by removalling eyeball. Blood was kept on ice until centrifugation (3,000 g, 10 min at 4°C), and the plasma was stored at -20°C until analysis. Blood triglyceride levels were determined by a commercially available assay kit (Sigma, Triglyceride—GPO-Trinder). All animals were killed by cervical dislocation. BAT and WAT depots were collected, weighed and quickly frozen in liquid nitrogen and stored at -80°C until further processed.

### Cell culture

C3H10T1/2 cells were purchased from Cell Bank of Type Culture Collection of China Science Academy (Shanghai, China) and grown in Dulbecco's modified Eagle's medium (DMEM) supplemented with 10% fetal bovine serum (FBS) (Gibco BRL) in a 5% CO_2_ incubator at 37°C. MEFs were prepared from 13.5-d embryos of the *SIRT1*^+/+^ *SIRT1*^−/+^ and *SIRT1*^−/−^ mice as reported elsewhere [37]. To induce adipocyte lineage commitment, C3H10T1/2 stem cells were plated at low density and cultured in DMEM containing 10% calf serum with resveratrol (25μM) or nicotinamide (10mM). After the cells reached postconfluence, they were induced to differentiate by using the standard 3T3-L1 differentiation protocol as previously described [38].

### Gene expression analysis

Total RNA was isolated from cells using TRIzol reagent following the protocol provided by the manufacturer (Invitrogen) and reverse transcribed according to the manufacturer's protocol (Takara, Dalian, China). Real-time PCR was performed by mixing cDNA with primers, and iTaq™ Universal SYBR^®^ Green Supermixquantitative PCR analysis reactions (Bio-Rad, USA). Real-time PCR was performed using a LightCycler^®^ 480 System with supplied software (Roche, USA), according to the manufacturer's instructions. RNA expression levels were compared after normalization to endogenous β-actin. The primer sequences used in this study are listed in Supplementary Table S1.

### Plasmids

Constitutively active *β-catenin* mutant (S33, 37, 45A T41A, *β-catenin mut*) was amplified from *β-catenin* (S33, 37, 45A T41A) cloning plasmid from Dr. Tony Kouzarides (University of Cambridge). pCMV-catenin and pCMV-catenin-mut were constructed by cloning the *β-catenin* and *β-catenin mut* fragments into pCMV-HA (Clontech), respectively [39]. Lentiviral shRNA constructs were generated in a pLKO.1 backbone (see Supplementary Table S2 for shRNA hairpin sequences). Mature virus was produced in HEK293T cells transfected with the pLKO.1 construct and psPAX2 and pMD.2G plasmids.

### Wnt signaling pathway PCR array

The Wnt Signaling Pathway RT^2^ Profiler PCR Array profiles the expression of 88 genes related to WNT-mediated signal transduction and 8 reference genes that are listed in Supplementary Table S3. The RT^2^ Profiler PCR arrays specific for the Wnt signaling pathway (CTB103) was purchased from ctbioscience and used according to manufacturer's instructions.

### Immunoblotting and immunoprecipitation

Whole-cell lysates of mammalian cells were prepared and analyzed for immunoblotting (IB) and immunoprecipitation (IP), as previously performed [40]. Whole-cell protein lysates were extracted with a solution containing 20 mM Tris-HCl (pH 7.5), 150 mM NaCl, 1% Triton X-100, 10 mM Na4P2O7, 1 mM Na3VO4, 2 mM EDTA, 0.5mM leupeptin, and 1 mM PMSF (Beyotime, China). The protein concentrations were determined using a Bradford Protein Assay Kit (Beyotime, China), and proteins were separated by 10% SDS-PAGE. The separated proteins were then transferred to polyvinylidene difluoride (PVDF) membranes (Bio-Rad, USA). Membrane blocking to prevent nonspecific binding was done with TTBS buffer (10 mM Tris-HCl (pH 7.6), 150 mM NaCl, 0.1% Tween) containing 5% skim milk powder. The blocked membranes were then incubated with a mouse specific anti-SIRT1 antibody (number 2028; Cell Signaling Technology Inc, Danvers, MA, USA), or an anti-PPARγ (number sc-1984; Santa Cruz), or an anti-FABP4 (number 2120; Cell Signaling Technology Inc.), or an anti-adiponectin (number 2789; Cell Signaling Technology Inc.), or an anti-β-catenin (number 9587; Cell Signaling Technology Inc.), or an anti-Cyclin D1 (number 2922; Cell Signaling Technology Inc.), or an anti-acetyl-Histone H3 (Lys9) (number 9671; Cell Signaling Technology Inc.), or an anti-acetyl-Histone H4 (Lys16) (number 13534; Cell Signaling Technology Inc.), or a rabbit polyclonal anti-TBP (number 8515; Cell Signaling Technology Inc.), and β-actin (number 3700; Cell Signaling Technology Inc.) for overnight at 4°C. Secondary antibodies were used according to the manufacturer's instructions. Secondary-antibody binding was detected using an enhanced chemiluminescence detection kit (Thermo Fisher Scientific, USA) according to the manufacturer's instructions. Protein levels were normalized to β-actin using Image J analysis software.

### SIRT1 activity assay

The deacetylase activity of SIRT1 was assessed by a fluorometric kit (Sigma, CS1040) in accordance with the instructions of manufacturer.

### ChIP assay

ChIP assays were performed according to the manufacturer's protocol (Upstate Biotechnology, Waltham, MA, USA). Chromatin was immunoprecipitated using rabbit anti-SIRT1 antibodies (number ab15830; Abcam). PCR was performed at a final template dilution of 1:50. The primer sequences used in this study are supplied in Supplementary Table S4.

### Luciferase reporter assay

The TOP/FOP Flash assays were performed according to the manufacturer's instructions. The cells were transfected with 1m g TOP Flash or 0.24mg FOP Flash plasmid (Millipore), with 0.1mg of renilla luciferase expression plasmid (Millipore) per well, using the Tfx-50 transfection reagent in a 24-well plate. The cells were treated as indicated, and luciferase activity was measured with the Dual-Luciferase reporter assay system (Promega).

### Oil red-O staining

Oil red O (Sigma-Aldrich) staining was performed as described previously [41]. Cells were washed twice with PBS and fixed with 10% formaldehyde for 45 minutes at room temperature. After washing with distilled water twice and 50% isopropanol once, the cells were stained for 1 hat room temperature with filtered Oil red O/60% isopropanol solution. The cells were washed twice with distilled water and twice with PBS. Adipocytes stained red were recorded by light microscopy (Leica German).

### Hematoxylin and eosin (H&E) staining

Fresh fat tissues were collected at 12 wk of age and fixed in 4% neutral buffered formalin solution (HT501; Sigma). The tissue slides were obtained through serial cross-section cutting at 8-μm thickness and processed with a standard procedure [13]. Then observed under optical microscope and imaging (Olympus Polaroid DMC-IE camera, Polaroid Corp., Waltham, MA).

### Statistical analysis

Means, SD, and SEM were analyzed using Microsoft Excel. t tests and one-way analysis of variance (ANOVA) tests were conducted, along with corresponding posttests, as indicated; Calculation of the average was performed using at least three biological replicas. *p* < 0.05 was considered significant. *p < 0.05, **p < 0.01.

## SUPPLEMENTARY MATERIALS FIGURE AND TABLES


