# Supplementary material for: SIRT1 suppresses adipogenesis by activating Wnt/β-catenin signaling in vivo and in vitro
Source: Oncotarget. 2016 Oct 20;7(47):77707–20. doi: 10.18632/oncotarget.12774 (PMC5363615; doi:10.18632/oncotarget.12774)
Supplement: Supplementary file 1 [file oncotarget-07-77707-s001.pdf]

SIRT1 suppresses adipogenesis by activating Wnt/β-catenin signaling in vivo and in vitro

SUPPLEMENTARY FIGURE AND TABLES

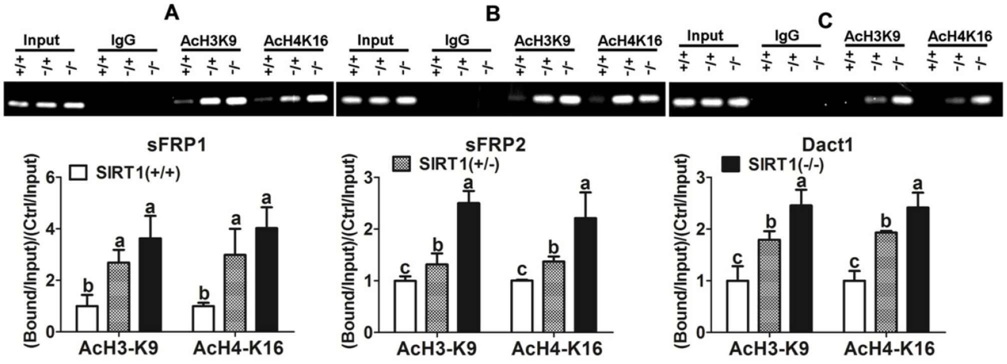

Supplementary Figure S1: Pooled populations of differentiated MEF cells were analyzed via ChIP. ChIP was performed with antibodies acetylatedH3K9 and H4K16, or with IgG antibody controls. Promoter sequence was amplified by RT-PCR under linear conditions for the genes *sFRP1* **A.** *sFRP2* **B.** and *Dact1* **C.** The acetylation of H3K9 and acetylation of H4K16 at the *sFRP1* (A), *sFRP2* (B) and *Dact1* (C) promoters were measured by Real-time PCR. Values are means ± SD. **P* < 0.05. n = 3 per group.

Supplementary Table S1: Primers for Realtime PCR assay

Gene	Primers (sense/antisense 5’-3’)	Product length (bp)	TM (°C)
PPARγ	TGGGTGAAACTCTGGGAGATTC<br>AGAGGTCCACAGAGCTGATTCC	250	60
ap2	GTGTGATGCCTTTGTGGGAAC<br>CCTGTCGTCTGCGGTGATT	235	60
Adiponectin	GCTCTCCTGTTCCTCTTAATCCT<br>CCAGTGCTGCCGTCATAATG	437	60
SIRT1	CCTGACTTCAGATCAAGAGACGGTA<br>CTGATTAAAAATGTCTCCACGAACAG	360	62
sFRP1	CACAACGTGGGCTACAAGAA<br>CTCGGGGAACTTGTCACATT	281	60
sFRP2	AATCGGCATCTAAGTCTT<br>GCAATGAGGAATGGTTAC	129	56
sFRP4	CACCACAGCACTCAGGAGAA<br>ACAGACTTGCAGGGCTTGAT	161	58
sFRP5	AGATGTGCTCCAGTGACTTT<br>CGTCAGGTTGTCTAACTGTG	171	60
Dact1	TCTGAGGAATGGAAGTGTG<br>GTCTGTCTTTGAGTCTTTGG	169	58
Dact2	GACTACGAGCCGCACTGG<br>GCAGGAGGTGGACAGAGAAC	341	60
Dact3	GCGCAGCTCAGGCTTCTATGAA<br>GCAGTGGGCTAGGTGTCAGGAA	323	60

(*Continued*)

Gene	Primers (sense/antisense 5'-3')	Product length (bp)	TM (°C)
Cyclin D1	AAATGCCAGAGGCGGATGAG<br>AAGAAAGTGCGTTGTGCGGT	200	60
c-Myc	TTCCCTACCCGCTCAACGACAG<br>TTGCCTCTTCTCCACAGACACCAC	227	61
β-actin	GGCACCACACCTTCTACAATG<br>GGGGTGTTGAAGGTCTCAAAC	133	60

Supplementary Table S2: Primers for RNAi plasmid construction

pLKO.1 Lentiviral Hairpin Sequences	sequence (5'to3')
sh-luciferase-F	CCGGCGCTGAGTACTTCGAAATGTCCTCGAGGACATTTCGAAGTACTCAGCGTTTTTG
sh-luciferase-R	AATTCAAAAACGCTGAGTACTTCGAAATGTCCTCGAGGACATTTCGAAGTACTCAGCG
sh-SIRT1-F	CCGGAGTGAGACCAGTAGCACTAATCTCGAGATTAGTGCTACTGGTCTCACTTTTTTG
sh-SIRT1-R	AATTCAAAAAAGTGAGACCAGTAGCACTAATCTCGAGATTAGTGCTACTGGTCTCACT
sh-sFRP1-F	CCGGGCTTGTGCTGTTCCTGAAGAACTCGAGTTCTTCAGGAACAGCACAAGCTTTTTG
sh-sFRP1-R	AATTCAAAAAGCTTGTGCTGTTCCTGAAGAACTCGAGTTCTTCAGGAACAGCACAAGC
sh-sFRP2-F	CCGGCGGCATCGAGTACCAGAACATCTCGAGATGTTCTGGTACTCGATGCCGTTTTTG
sh-sFRP2-R	AATTCAAAAACGGCATCGAGTACCAGAACATCTCGAGATGTTCTGGTACTCGATGCCG
sh-Dact1-F	CCGGGCATCTGGTGAAAGCTCAGTTCTCGAGAACTGAGCTTTCACCAGATGCTTTTTG
sh-Dact1-R	AATTCAAAAAGCATCTGGTGAAAGCTCAGTTCTCGAGAACTGAGCTTTCACCAGATGC

Supplementary Table S3: Oligo Wnt signaling pathway microarray

	A	*B*	*C*	*D*	*E*	*F*
1	Fbxw4	Ccnd3	Csnk2a1	Ctbp1	Ctbp2	Dlk1
2	Nlk	Kat2b	Pitx2	Ppp2ca	Wisp1	Fbxw2
3	Csnk1a1	Sirt1	Fbxw11	Csnk1d	Csnk1g1	Dact2
4	Rb1	Apc	Axin1	Bmp4	Ctnnb1	Ccnd1
5	Fzd1	Fzd3	Fzd4	Fzd5	Fzd6	Fzd7
6	Lrp6	Myc	Sfrp2	Sfrp1	Frzb	Sfrp4
7	Wnt11	Wnt2	Wnt2b	Wnt3	Wnt4	Wnt5a
8	Dkk3	Sfrp5	Gsk3b	Dkk2	Fzd2	Pygo1
9	E2f1	Fgf4	Fosl1	Fshb	Aes	Actb
10	Ppp2r1a	Dact1	Sirt2	Ctnnbip1	Senp2	B2m
11	Cxxc4	Dixdc1	Dact3	Wnt3a	Foxo1	Hprt1
12	Ccnd2	Dkk1	Dvl1	Dvl2	Dvl3	Gapdh
13	Fzd8	Foxn1	Jun	Lef1	Lrp5	Rpl27
14	Sox17	Wnt8a	Tcf3	Wnt1	Wnt10a	Oaz1
15	Wnt5b	Wnt6	Wnt7a	Wnt7b	Wif1	PPC
16	Wnt16	Wnt9a	Dkk4	Ep300	Gsk3a	GDC

Supplementary Table S4: Primers for ChIP assay

Gene	Primers (sense/antisense 5'-3')	Product length (bp)	TM (°C)
sFRP1	GTAAACCGATCCTTCTGGC<br>CTGCACCTACTTGCGACG	171	58
sFRP2	CCAGAAAGTAGTGACCGG<br>GAGCAGAGTGAGCAGAGG	134	58
Dact1	ACAAAGGCGACCTCCAAA<br>TCCTCTCCCTTGGCTCTG	134	58

Supplementary Table S5: Effect of Wnt signalling by Wnt RT-qPCR array with Res

Gene	fold change	*P* value	Gene	fold change	*P* value
Foxn1	-9.46	0.00	*Wnt2*	-1.66	0.39
Wnt4	-6.18	0.00	*Dixdc1*	-1.65	0.25
Fzd1	-5.93	0.02	*Cxxc4*	-1.60	0.11
Dkk2	-4.69	0.01	*Wnt5b*	-1.57	0.20
Sfrp4	-4.40	0.12	*Gsk3b*	-1.56	0.05
Wnt11	-3.66	0.01	*Csnk1d*	-1.49	0.01
Ctnnb1	-3.36	0.05	*Ctbp2*	-1.39	0.25
Fzd4	-3.01	0.07	*Senp2*	-1.32	0.55
Ppp2ca	-2.72	0.01	*Ctnnbip1*	-1.31	0.02
Dact1	-2.68	0.01	*Pygo1*	-1.29	0.12
Sfrp1	-2.49	0.00	*Ccnd1*	-1.27	0.33
Sfrp2	-2.48	0.03	*Foxo1*	-1.24	0.14
Rb1	-2.48	0.12	*Gsk3a*	-1.22	0.85
Fzd8	-2.38	0.11	*Csnk1g1*	-1.21	0.52
Lrp6	-2.35	0.05	*Csnk1a1*	-1.18	0.60
Fzd7	-2.32	0.12	*Ep300*	-1.18	0.12
Fzd5	-2.21	0.03	*Ccnd3*	-1.18	0.71
Ppp2r1a	-2.19	0.29	*Aes*	-1.15	0.65
Fbxw2	-2.13	0.00	*Lrp5*	-1.04	0.63
Axin1	-2.12	0.13	*Lef1*	-1.03	0.78
Ctbp1	-1.99	0.14	*Nlk*	-1.02	0.81
Frzb	-1.97	0.05	*Wisp1*	1.00	0.95
Kat2b	-1.96	0.42	*Fzd2*	1.06	0.94
Dvl3	-1.94	0.05	*Sirt2*	1.09	0.82
Fzd3	-1.92	0.00	*Dlk1*	1.14	0.77
Dvl2	-1.81	0.03	*Jun*	1.15	0.27
Fbxw4	-1.78	0.05	*Sirt1*	1.24	0.59
Dact3	-1.77	0.00	*E2f1*	1.48	0.22
Tcf3	-1.72	0.11	*Myc*	1.64	0.06
Pitx2	-1.71	0.01	*Dkk3*	2.62	0.00
Fbxw11	-1.68	0.05	*Bmp4*	2.93	0.02
Csnk2a1	-1.66	0.07			

Note: The fold change compared with control group. – Means down regulation. Detection of cycle number more than 35 cycle gene was not listed.

Supplementary Table S6: Effect of Wnt signalling by Wnt RT-qPCR array with Nico

Gene	fold change	*P* value	Gene	fold change	*P* value
Ctnnb1	-4.37	0.04	*Fbxw2*	-1.52	0.52
Fzd1	-4.21	0.03	*Sirt1*	-1.51	0.37
Myc	-3.84	0.02	*Dact3*	-1.49	0.01
Wnt4	-3.74	0.00	*Wisp1*	-1.43	0.76
Kat2b	-3.39	0.04	*Fbxw11*	-1.42	0.81
Ppp2ca	-3.39	0.01	*Fzd7*	-1.23	0.50
Gsk3b	-3.34	0.01	*Wnt2*	-1.19	0.75
Sfrp4	-3.29	0.17	*Aes*	-1.16	0.77
Dvl3	-2.81	0.03	*Wnt6*	-1.12	0.67
Rb1	-2.52	0.25	*Dact2*	-1.09	0.90
Ppp2r1a	-2.51	0.36	*Wnt1*	-1.09	0.90
Ccnd1	-2.43	0.04	*Wnt5a*	-1.09	0.90
Fzd2	-2.42	0.15	*Fbxw4*	-1.04	0.58
Lef1	-2.37	0.00	*Foxo1*	1.02	0.91
Jun	-2.29	0.05	*Wnt5b*	1.04	0.88
Dvl2	-2.13	0.04	*Nlk*	1.07	0.74
Axin1	-2.07	0.12	*Dlk1*	1.12	0.81
Dixdc1	-2.00	0.38	*Ccnd3*	1.14	0.82
Ctbp1	-1.98	0.62	*Senp2*	1.18	0.74
Dkk2	-1.95	0.07	*Wnt11*	1.19	0.56
Sirt2	-1.94	0.07	*Fzd3*	1.22	0.34
Tcf3	-1.94	0.07	*Gsk3a*	1.32	0.06
Fzd5	-1.74	0.06	*Frzb*	1.45	0.04
Csnk1d	-1.74	0.02	*Csnk1a1*	1.53	0.04
Sfrp1	-1.69	0.03	*Cxxc4*	1.54	0.13
Lrp6	-1.67	0.16	*Foxn1*	1.77	0.02
Csnk2a1	-1.61	0.14	*Fzd4*	1.82	0.06
Csnk1g1	-1.59	0.25	*Sfrp2*	2.24	0.00
Ctbp2	-1.57	0.83	*Dact1*	2.84	0.03
Ctnnbip1	-1.53	0.02	*Wnt3*	3.41	0.38

Note: The fold change compared with control group. – Means down regulation. Detection of cycle number more than 35 cycle gene was not listed.
